# Cell Differentiation Degree as a Factor Determining the Role for Different T-Helper Populations in Tuberculosis Protection

**DOI:** 10.3389/fimmu.2019.00972

**Published:** 2019-05-08

**Authors:** Irina Lyadova, Irina Nikitina

**Affiliations:** ^1^Laboratory of Cellular and Molecular Mechanisms of Histogenesis, Koltsov Institute of Developmental Biology, Moscow, Russia; ^2^Laboratory of Biotechnology, Department of Immunology, Central Tuberculosis Research Institute, Moscow, Russia

**Keywords:** tuberculosis, Th1, Th17, non-classical Th1, correlates of protection

## Abstract

Efficient tuberculosis (TB) control depends on early TB prediction and prevention. Solution to these tasks requires knowledge of TB protection correlates (TB CoPs), i.e., laboratory markers that are mechanistically involved in the protection and which allow to determine how well an individual is protected against TB or how efficient the candidate TB vaccine is. The search for TB CoPs has been largely focused on different T-helper populations, however, the data are controversial, and no reliable CoPs are still known. Here we discuss the role of different T-helper populations in TB protection focusing predominantly on Th17, “non-classical” Th1 (Th1^*^) and “classical” Th1 (cTh1) populations. We analyze how these populations differ besides their effector activity and suggest the hypothesis that: (i) links the protective potential of Th17, Th1^*^, and cTh1 to their differentiation degree and plasticity; (ii) implies different roles of these populations in response to vaccination, latent TB infection (LTBI), and active TB. One of the clinically relevant outcomes of this hypothesis is that over-stimulating T cells during vaccination and biasing T cell response toward the preferential generation of Th1 are not beneficial. The review sheds new light on the problem of TB CoPs and will help develop better strategies for TB control.

## Introduction

Tremendous efforts have been made to improve tuberculosis (TB) control. Nevertheless, TB remains a global public health threat. Multi-drug resistance of *M. tuberculosis* (*Mtb*), HIV infection, malnutrition, aging and increased usage of immune suppressant drugs contribute to TB spread (1–4). Most of these factors operate by altering host immune resistance to various infections, including TB. In these settings, evaluating the individual level of TB protection and TB risk and developing effective vaccines to increase population resistance are important tasks. Their solutions require knowledge of TB protection correlates (TB CoPs) (5).

Multiple cellular populations and molecular pathways mediate antimycobacterial immunity (6–14). Among them, IFN-γ-producing CD4 lymphocytes play the major role (6–9). Consequently, Th1 lymphocytes have long been regarded as TB CoPs. However, recent data do not fully support this concept. Other populations of Th lymphocytes have appeared as candidate TB CoPs. Yet, the data are controversial, and no reliable TB CoPs are still known. Here we analyze recent data and discuss protective potential of “classical” Th1 (cTh1), “non-classical” Th1 (Th1^*^), and Th17 lymphocytes during latent TB infection (LTBI), active TB and following vaccination.

## The Multifaceted Face of TB Protection and Its Correlates

Mechanistic CoPs are defined as immune parameters directly responsible for host protection. In contrast, non-mechanistic CoPs (or biomarkers) are determined as markers that correlate with the protection but are not causally responsible for it (15, 16). Protection can be determined in different ways, i.e., as host ability to (i) prevent the acquisition of the infection; (ii) clear the pathogen after the initial onset of the infection; (iii) limit pathogen replication and maintain the infection in inactive (latent) form; or (iv) limit disease progression and severity. CoPs against *Mtb* infection, TB disease and TB disease progression/severity may differ.

Knowledge of TB CoPs is important for estimating the individual level of protection and developing and testing new vaccines. “Individual” and vaccine CoPs may differ.

The most common approaches to identify individual TB CoPs rely on: (i) the assessment of *Mtb* infection severity in immunologically-manipulated mice; (ii) the analysis of immune response and TB pathology in other animal models, including non-human primates (NHP) and bovine models [reviewed in (17–20)]; (iii) the comparison of immune responses in poorly and well-protected individuals, primarily in TB patients and LTBI subjects, and in TB patients with diverse TB severity. At the bottom, these approaches address CoPs against TB disease and TB severity (or their experimental surrogates), but do not measure CoPs against *Mtb* infection. The latter is difficult to address due to the lack of adequate animal models and methods to evaluate *Mtb* persistence and clearance in humans (19).

*In the vaccination field*, experimental studies allow us to directly compare vaccine immunogenicity and protectivity (21–24), whereas clinical evaluation of vaccine protectivity is difficult, limiting many studies to the analysis of vaccine immunogenicity only.

In the review we mainly focus on mechanistic CoPs paying attention to delineate individual and vaccine CoPs, and models used for their detection. We focus predominantly on blood CoPs due to their clinical relevance and limited number of studies addressing tissue-associated CoPs in humans.

## Th1 Lymphocytes

Th1-response magnitude is often used as a measure of TB protection and vaccine immunogenicity (7–9, 11, 13, 19, 25–28). The concept relies on observations showing that failure to develop Th1 response increases severity of experimental *Mtb* infection in mice and TB risk in humans. Mice deficient in CD4 lymphocytes, IFN-γ or other type 1 response genes develop extremely severe TB (29–32). Patients with AIDS and patients receiving anti-TNF therapy have increased TB risk (4, 33–35). Children bearing mutations in the genes of IL-12/IFN-γ axis exhibit Mendelian susceptibility to mycobacterial diseases (44–46). Nevertheless, the fact that Th1 are needed for *Mtb* control does not signify that their magnitude reflects the degree of protection (11, 16, 47–49). Reports on the lack of correlation between Th1 and protection have been accumulated.

### Experimental Studies

Mouse IFN-γ^−/−^ CD4^+^ cells provided protection against *Mtb in vitro* (50) and following adaptive transfer *in vivo* (51, 52); *in vivo*, hyper-production of IFN-γ was deleterious (53). *Vaccination* of mice with BCG stimulated Th1, however the response did not reflect the strength of protection (21, 54). To enhance BCG-induced antimycobacterial immunity, prime-boost strategies were suggested. In mice and NHP, boosting strengthened Th1 response (55–59), but in most studies this did not correlate with protection (55–57).

### Clinical Studies

In humans, LTBI and active TB serve as surrogates of effective and ineffective protection, respectively. Comparative analyses of Th1/IFN-γ during LTBI and TB have given inconsistent results on whether the responses are higher during LTBI or TB (60–66). Interferon-gamma release assays (IGRA) do not discriminate between LTBI and active TB, indicating that most TB patients do not exhibit Th1 deficiency (67, 68). In TB patients, disease severity does not correlate with diminished Th1/IFN-γ; patients with active disease develop higher *Mtb*-specific IFN-γ responses compared to patients with residual TB lesions (64, 65, 69).

While the magnitude of Th1 lymphocytes does not correlate with protection and does not differentiate between LTBI and TB, Th1 activation and differentiation allow distinguishing LTBI and TB. Specifically, *Mtb*-specific Th1 persisting during LTBI are significantly less activated, less differentiated and contain fewer cycling lymphocytes compared to Th1 circulating during TB (evaluation based on the expression of CD27, HLA-DR/CD38, and Ki67) (70–74). It remains, however, unclear whether low-activated/differentiated Th1 are mechanistically involved in LTBI maintenance or whether their predominance during LTBI simply reflects low infection activity.

*In vaccine clinical studies*, BCG and new candidate vaccines appeared as potent inducers of Th1. However, in most studies, Th1 response did not coincide with vaccine efficacy. In one recent study, BCG-induced IFN-γ-secreting T cells associated with reduced TB risk in South Africa infants (75). However, a previous study in the same population found no association of BCG-specific Th1 with TB risk (76). Similarly, BCG-boost strategies and attenuated vaccines enhanced Th1 response (77–80), but did not show satisfactory protection in clinical testing [(77, 78, 81), reviewed in (19, 27, 82)].

Overall, the levels of Th1 immunity reflect the strength of *Mtb* infection rather than the degree of protection and do not correlate reliably with vaccine efficacy.

## Th17 Lymphocytes

### Experimental Studies

Th17 are pleiotropic cells with neutrophil-stimulating and pro-inflammatory activities (49, 83, 84). As neutrophils had been implicated in TB pathology (47, 85, 86), Th17 were initially thought to promote TB progression. However, in most experimental studies they conferred protection (87–95).

In mice infected with *Mtb*, Th17 promoted granulomatous response, participated in the formation of B-cell follicles and activated macrophages for *Mtb* control (87–90). *Vaccination of mice and NHP* with BCG or subunit vaccines induced both Th17 and Th1 (56, 96, 97). However, in a mouse model, only Th17 were essential for vaccine efficacy (56). Of note, protection provided by Th17 was mediated through the enhanced generation of Th1 and their higher accumulation in the lung tissue (91–94). The data point to Th17 as mediators of vaccine-induced protection but raise a question on why Th1 themselves do not mark vaccine efficacy if they are needed to mediate Th17-dependent protection.

### Clinical Studies

The results of clinical Th17/IL-17 analyses are ambiguous. Several, but not all studies reported associations between TB susceptibility and polymorphisms in genes encoding IL-17 (98, 99). Transcriptional and clinical analyses of healthy adolescents in South Africa revealed an association between inhibited Th17 responses and TB development (100). However, comparison of Th17/IL-17 levels in LTBI subjects and TB patients yielded inconsistent results: some studies reported heightened Th17/IL-17 levels in LTBI subjects (101–103), others demonstrated increased Th17/IL17 in TB patients (104–107), some did not reveal differences between LTBI and TB groups (108) or found extremely low frequencies of *Mtb*-specific Th17 in both groups (109, 110). In our study, *Mtb*-specific Th17 were rare in the blood, but readily identifiable in the lungs of TB patients (110). However, it remained unclear whether lung-residing Th17 contributed to TB protection or pathology, since there was no possibility to measure lung Th17 in well-protected individuals.

*In vaccine clinical studies*, BCG and new candidate vaccines induced IL-17 producing cells. Yet, the cells either did not correlate with the risk of TB [BCG (76)] or their role remained uncertain [MVA85A (77)].

In summary, Th17 are involved in vaccine-induced protection in mice. Their role in vaccine-induced immunity in humans as well as in immune protection and pathology during *Mtb* infection (in both humans and experimental animals) remains uncertain.

## Polyfunctional Th1 and Th17 Lymphocytes

Th1 and Th17 populations are heterogeneous and contain subpopulations with diverse cytokine profiles.

### Polyfunctional Th1 Lymphocytes

*In experimental studies*, polyfunctional IFN-γ^+^TNF-α^+^IL-2^+^ Th1 lymphocytes (PFL) were analyzed using murine, bovine and NHP models. In all of them, BCG and novel TB vaccine candidates elicited PFL, however, not in all studies correlative relationships between vaccine-induced PFL and protection were found [reviewed in details in (15)].

*In clinical studies*, many groups associated LTBI maintenance with the persistence of PFL (15, 65, 111–114), which was attributed to their low-differentiation degree and central memory characteristics (15). Yet, some groups found more PFL in TB patients (115–117) or did not find differences between LTBI and TB (106). Within the group of TB patients, PFL did not correlate with TB severity (65). In humans, BCG, subunit, recombinant protein and viral vector vaccines induced PFL; data on their correlation with vaccine efficacy are limited to BCG and BCG-prime/MVA85A-boost; both did not correlate with decreased TB risk [68, 69].

Recently, Orlando and co-authors described a new population of polyfunctional *Mtb*-responding CD4 human lymphocytes, T_CNP_ (118, 119). T_CNP_ produced IFN-γ, TNF-α, and IL-2 but expressed CD45RA^+^CCR7^+^ naïve-like phenotype. The magnitude of T_CNP_ reflected TB activity. It was suggested that the cells present potential target for vaccination and immunotherapeutic strategies. However, more studies are needed to understand T_CNP_ differentiation, function and link to *Mtb* infection activity.

### Th17.1 Lymphocytes

Th17.1 co-produce IFN-γ/TNF-α and IL-17, co-express T-bet and RORγt and differentiate from Th17 in the presence of IL-12 and inflammatory cytokines, primarily IL-1β (120–122). During autoimmune diseases, Th17.1 are hyperpathogenic (49). During TB, Th17.1 are detected in the brochoalveolar fluid (123) and lungs (110), but are rare in blood (110, 124), leaving their role in TB uncertain.

### Regulatory Th17

Regulatory Th17 co-produce IL-17 and IL-10. Recently, the enrichment of IL10^+^Th17 lymphocytes during LTBI has been reported (123). The data correspond to the beneficial, inflammation-limiting role of Treg demonstrated in NHP model of TB (125) and suggest IL10^+^Th17 as a new CoP (123). Yet, more studies are needed to support this conclusion.

Overall, the induction of Th1 PFL is not sufficient or even necessary for TB protection (15). The role of other polyfunctional populations remains to be established.

## Non-classical Th1 Lymphocytes

T-helper populations are categorized based on cytokine profiles, transcriptional regulation and priming requirements (36, 37, 126, 127). Another categorization principle relies on the expression of chemokine receptors. In humans, the expression of CXCR3, CCR4, and CCR6 divides T cells into CXCR3^+^CCR4^−^CCR6^−^, CXCR3^−^CCR4^+^CCR6^−^, and CXCR3^−^CCR4^−^CCR6^+^ subsets that correspond to Th1, Th2, and Th17, respectively (128, 129). CXCR3 is a marker of Th1, CCR6 is a marker of Th17 and their progeny. Recently, a population of non-classical Th1 (Th1^*^ or ex-Th17) has been described. Th1^*^ produce IFN-γ in the absence of IL-17, but express CXCR3^+^CCR6^+^ phenotype (128). A few studies have demonstrated Th1^*^ enrichment during LTBI and have suggested them as a new TB CoP (130–133). Nevertheless, it remains unclear why protection should correlate specifically with Th1^*^ if functionally Th1^*^ and cTh1 are similar.

## Differences and Relationships Between cTh1, Th1^*^ and Th17 During TB

### Memory and Effector Populations of CD4^+^ Lymphocytes

Besides being divided into different Th populations based on cytokine profiles, CD4^+^ lymphocytes are divided into several clusters based on cell phenotype, differentiation, and homing properties. CD4^+^ lymphocytes circulating in human blood in the quiescent state are classified into T_N_, T_SCM_, T_CM_, T_TM_, T_EM_, and T_TE/_T_EMRA_ clusters [(Table 1); (40, 119, 134–140)]. According to the lineage differentiation model, CD4^+^ cells progressively differentiate along these clusters, with less differentiated cells being longer-lived, having higher multipotency and higher self-renewal and protective capacities compared to more differentiated cells (138, 141–143).

**Table 1:**
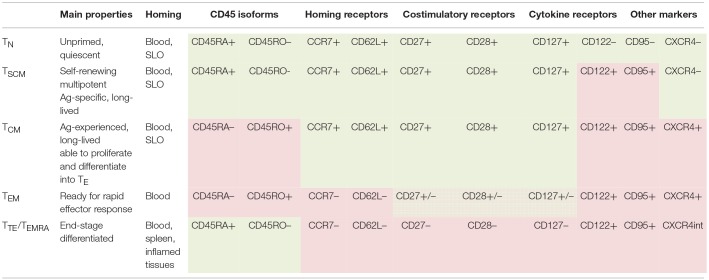
Functional and phenotypic characteristics of naïve and memory populations of CD4^+^ human lymphocytes (40, 118, 134–139).

*SLO, secondary lymphoid organs; T_N_, naïve T-cells; T_SCM_, stem cell memory T cells; T_CM_, central memory T cells; T_EM_, effector memory T cells; T_EM_/T_EMRA_, end-stage differentiated T cells. Green, phenotype characteristic for (shared with) naïve lymphocytes. Pink, phenotypes that differ from those of naïve lymphocytes. Checked, heterogeneous expression of markers by different subsets of the indicated population*.

Following the interaction with the cognate antigen, naïve and memory cells differentiate into effector lymphocytes (140). Depending on the strength and duration of antigenic stimulation, effector cells reach different differentiation states, which can be delineated by cell phenotype (137, 140, 144–148). Particularly, early-differentiated CD4^+^ effectors are CD27^+^CD28^+^, late-differentiated effectors are CD27^−^CD28^+^, exhausted effectors are CD27^−^CD28^−^; terminally-differentiated effectors are CD27^−^CD28^−^ and express CD57, other NK-cell and inhibitory receptors (140, 145). Along with their differentiation, effector CD4^+^ cells progressively decrease proliferative and survival potentials and increase cytokine secreting activity, with the exception of the exhausted and terminally-differentiated populations that decrease/lose cytokine production (137, 140, 145). Following antigen/pathogen clearance, most effectors die, whereas some give rise to memory populations. The less differentiated an effector cell is, the higher is its capacity to acquire memory state.

Overall, there is a link between T-cell stemness and memory potential and there are reciprocal relationships between these parameters and the strength of cell effector activity.

### Th1^*^ and cTh1 Display Phenotypic and Differentiation Differences

Considering that T-cell protective potential depends on cell differentiation, we have recently compared the differentiation states of Th1^*^ and cTh1 persistent during TB (110). Th1^*^ were less-differentiated: they contained more CD27^+^ cells, did not contain CD27^−^CD28^−^ cells and expressed T-bet at a lower level than cTh1 [a sign of memory cell precursors (134)]. We also found out that CXCR3^+^CCR6^+^ Th1^*^ cells stimulated *in vitro* with anti-CD3/CD28 antibodies, differentiated into CXCR3^+^CCR6^−^ cTh1-like lymphocytes, but not vice versa (110). Other authors reported a higher expression of anti-apoptotic protein Bcl-2 by Th1^*^ cells (41) and an overlap between the gene signatures of Th1^*^ and memory CD4^+^ cells persistent during LTBI (132). There are also overlaps between Th1^*^ and *Mtb*-specific T_SCM_, as both are CD27^+^, express CXCR3 and CCR6 and produce type 1 cytokines (149). Thus, compared to cTh1, Th1^*^ are more memory-biased, which, as we suppose, determines their role in LTBI maintenance. Of note, most TB studies identified Th1 based on intracellular IFN-γ, i.e., they did not distinguish between cTh1 and Th1^*^. This could account for a poor correlation between Th1 and TB protection.

Additional mechanism providing Th1^*^ with increased protective potential may lie in their expression of CCR6, as CCR6 participates in cell homing to the inflamed tissues (150, 151). Peripheral localization is critical for TB protection (152). Interestingly, tissue-resident memory cells (T_RM_) exhibit superior protective potential (153) and co-express CXCR3 and CCR6 (154).

Overall, although Th1^*^ and cTh1 exhibit similar functional activity in terms of the secretion of type 1 cytokines, Th1^*^ are less-differentiated, have higher survival and mucosal tissue homing capacities and higher protective potential.

### Lineage Relationships Between Th17, Th1^*^, and cTh1 Populations: Data and Hypotheses

Some authors suggest that Th1^*^ may originate from naïve precursors (40), yet multiple observations speak in favor of Th1^*^ generation from Th17 (40, 41, 155, 156). The Th17 → Th1 differentiation provides a mechanism to maintain long-lasting Th1 response *in vivo*. Indeed, cTh1 are highly differentiated and have poor persistent capacity (38). In contrast, Th17 have self-renewal properties similar to those of T_SCM_ (157), they are long-lived, plastic (38, 40) and acquire IFN-γ production in the presence of IL-12 and pro-inflammatory cytokines, primarily IL-1β (39, 42, 43, 158). *Mtb* have evolved multiple mechanisms to avoid host protective immunity, including the inhibition of IL-12 (159, 160). Yet, innate immune cells produce IL-12 and IL-1β during *Mtb* infection (161, 162). This creates conditions necessary for the differentiation of vaccine-induced Th17 into Th1^*^. Indeed, in mice vaccine-induced Th17 adapted Th1 characteristics following *Mtb* challenge (158). In a mouse model of autoimmune disease, Th17 comprised two subsets, CD27^+^ stemness-associated and CD27^−^/T-bet^+^ able to trans-differentiate into Th1-like cells (163). The data support Th17 → Th1 differentiation and the dichotomy between stemness and Th1-like properties.

**Figure 1:**
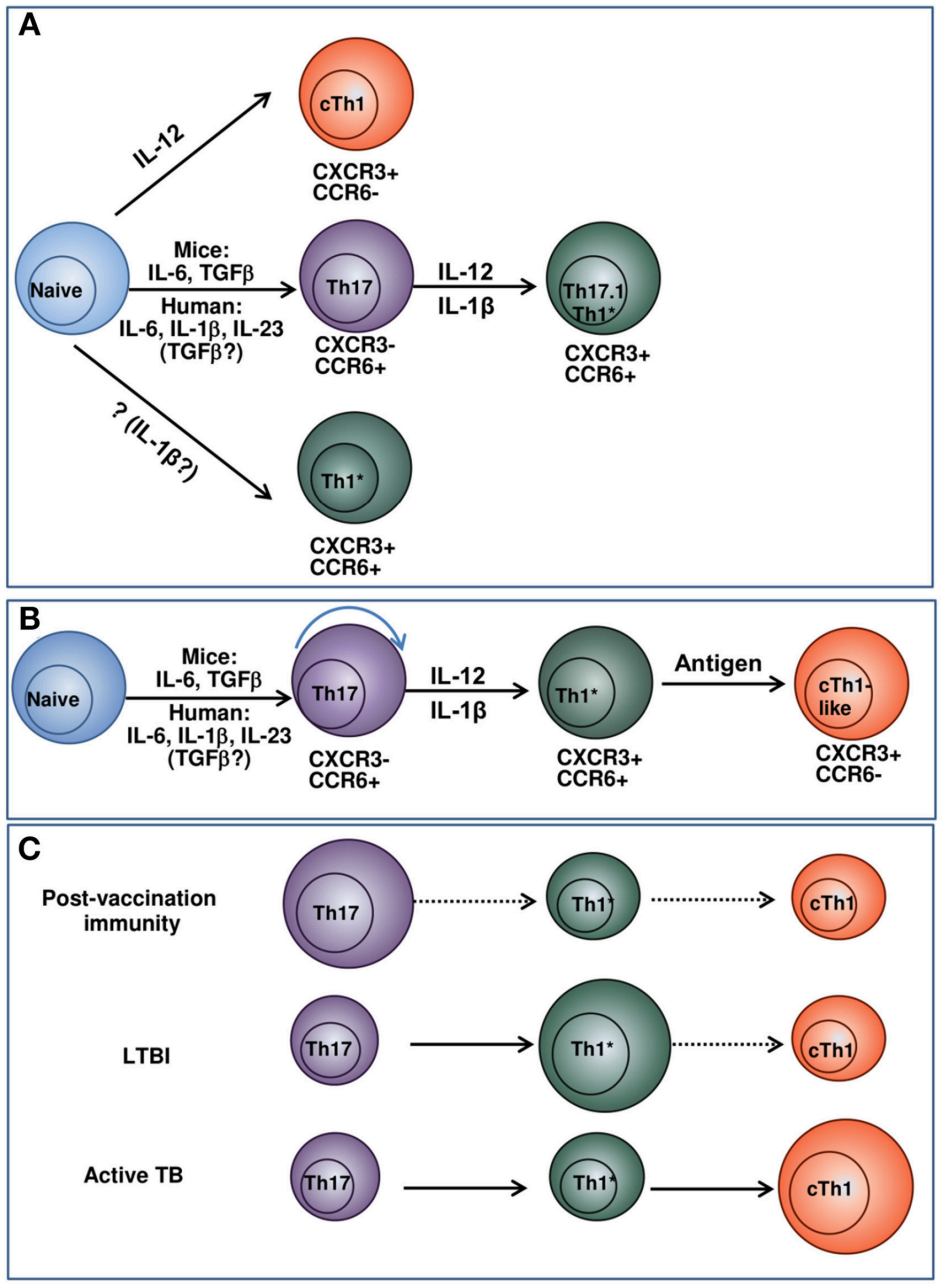
Model suggesting the relationships between Th17, Th1* and cTh1 populations and their potential roles in post-vaccination immunity, LTBI and active TB. **(A)** Current concepts of cTh1, Th17, and Th1* differentiation. cTh1 differentiate from naïve lymphocytes in the presence of IL-12 (36, 37). Th17 differentiate from naïve lymphocytes in the presence of cytokine mixture. In general, the differentiation of mouse Th17 cells depends on IL-6/TGF-β; the generation of human Th17 is driven by IL-23/IL-1β/IL-6. The involvement of TGF-β in the generation of human Th17 has been suggested by some authors, especially at low cytokine doses (36, 38, 39). When exposed to IL-12, IL-1β, or/and TNF-α, Th17 convert into IFN-γ producing Th17.1 and Th1* lymphocytes (38, 40–43). Alternatively, Th1* may derive directly from naïve lymphocytes under the action of cytokines that have not yet been identified (40). The concept considers Th1* and cTh1 as independent lineages of CD4^+^ T-cell differentiation. **(B)** Suggested pathway of cTh1, Th17, and Th1* differentiation. Naive cells progressively differentiate into Th17, Th1* and cTh1/cTh1-like lymphocytes. The depth of the differentiation depends on the strength of antigenic stimulation and cytokine milieu and is different in response to vaccination, LTBI and active TB. **(C)** Suggested pathway of cTh1, Th17, and Th1* differentiation and the predominance of different Th populations following vaccination, during LTBI and active TB. In response to vaccination, different populations of Th cells generate. Of them, Th17 have higher survival capacity and persist longer. Following *Mtb* infection, Th17 are exposed to antigen, IL-12 and pro-inflammatory cytokines and differentiate into IFN-γ producing Th1* CXCR3^+^CCR6^+^ cells. The cells persist during LTBI and maintain protection against TB disease. cTh1 are generated in response to vaccination, LTBI and TB disease, but do not persist for a long time. During active TB, their magnitude increases due to their permanent generation from naïve lymphocytes and/or Th1*. The size of the circles indicates the relative prevalence and protective roles of the corresponding subsets in the indicated conditions. For each condition, prevalent pathways of T-cell differentiation are indicated in solid arrows, otherwise dashed arrows are used.

Our data on a lower differentiation degree of Th1^*^ and their ability to transform into CXCR3^+^CCR6^−^ cells suggest Th1^*^ → cTh1 transition and the existence of Th17 → Th1^*^ → cTh1 differentiation pathway. Because T-cell differentiation is driven by antigenic and cytokine stimulation, the hypothesis implies that different populations dominate and mediate protection following vaccination, during LTBI and active TB (Figure 1). We suppose that Th17 persist in low-inflammatory conditions, maintain vaccine-induced protection and early response to *Mtb*; Th1^*^ are generated and support long-lasting protection during LTBI; cTh1 originate from naïve and/or Th1^*^ precursors during active TB. Certainly, more studies are needed to prove the hypothesis. However, it explains some existing data and controversies, such as: (i) the preferential link of vaccine-induced protection to Th17; (ii) a need for Th1 for the protection against TB along with the lack of correlation between post-vaccination Th1 and vaccine-induced protection; (iii) the predominance of Th1^*^ during LTBI and their contraction during active TB (discussed above). Important outcomes of the hypothesis, if it is supported, are: (i) in different conditions, protection is associated with different Th populations, i.e., there is no single T-cell-associated TB CoP; (ii) over-stimulating T cells during vaccination and biasing host response toward the preferential generation of Th1 (the aim of many current vaccination-boost strategies) are not beneficial.

## Closing Summary

Multiple studies have searched for TB CoPs, but the data are contradictory, many questions remain unanswered, and no reliable CoPs have been identified. Particularly, it is not clear why vaccine-induced pre-challenge Th1 do not correlate with protection, why Th17 better correlate with protection compared to Th1, and why LTBI maintenance is associated with Th1^*^ but not cTh1. The review addresses these questions by analyzing Th17, Th1^*^, and cTh1 properties and differentiation pathways. We suggest a hypothesis that links Th protective potential to cell differentiation degree, longevity and plasticity. We suppose that following the exposure to *Mtb*-antigens, Th cells transit along the Th17 → Th1^*^ → cTh1 pathway. The differentiation depth depends on the strength of antigenic stimulation, which is different following vaccination, during LTBI and active TB. Thus, under different conditions, immunotherapy and vaccination strategies should target different populations, and over-stimulating T cells during vaccination is not beneficial. Although more studies are needed to confirm the suggested assumptions, the hypothesis sheds new light on TB CoPs and should help develop better TB control strategies.

## Author Contributions

IL analyzed the literature and wrote the manuscript. IN contributed to manuscript writing.

### Conflict of Interest Statement

The authors declare that the research was conducted in the absence of any commercial or financial relationships that could be construed as a potential conflict of interest.
